# Transthyretin Amyloidosis—One of the Causes of Heart Failure in Patients with Severe Clinical Course of COVID-19

**DOI:** 10.3390/ijms26199806

**Published:** 2025-10-09

**Authors:** Zarina Gioeva, Liudmila Mikhaleva, Nikita Gutyrchik, Nikolay Shakhpazyan, Valentina Pechnikova, Konstantin Midiber, Andrej Kontorshchikov, Elizaveta Zentsova, Lev Kakturskij

**Affiliations:** 1 Avtsyn Research Institute of Human Morphology of Federal State Budgetary Scientific Institution, “Petrovsky National Research Centre of Surgery”, 117418 Moscow, Russia; mikhalevalm@yandex.ru (L.M.); gyt94@yandex.ru (N.G.); nshakhpazyan@gmail.com (N.S.); valiagtx@yandex.ru (V.P.); andreistr.ru@mail.ru (A.K.); liza-zentsova@mail.ru (E.Z.); levkaktur@mail.ru (L.K.); 2 Institute of Medicine, Peoples’ Friendship University of Russia, 117198 Moscow, Russia

**Keywords:** amyloidosis, heart failure, cardiosclerosis, transthyretin amyloidosis, COVID-19, immunohistochemistry

## Abstract

Wild-type transthyretin amyloidosis is an underdiagnosed condition that significantly contributes to mortality in the elderly population. This histopathological study describes autopsy findings in patients with severe clinical course of COVID-19 and ATTR not identified during life. Autopsy findings in the myocardium were analyzed in 19 patients with pre-existing ATTR who died from severe COVID-19. RT PCR was used for pre- and post-mortem detection of SARS-CoV-2 RNA. Immunohistochemical typing was performed with a broad panel of antibodies against different amyloid types. Autopsy specimens from the myocardium and lungs were positive for SARS-CoV-2 RNA in 10 (53%) cases. Microscopic examination of the myocardium revealed focal cardiosclerosis and cardiomyocyte dissociation in 15 (68%) cases, hypertrophy and atrophy of cardiomyocytes in 17 (77%) and 7 (32%), respectively, and myocarditis in 4 (18%) cases. Immunohistochemical analysis determined ATTR amyloidosis in all cases. In patients with rapidly progressive heart failure, the postmortem examination revealed multiple sites of interstitial amyloid deposits and focal cardiosclerosis in the myocardium. Pre-existing cardiac amyloidosis contributes to the aggressive clinical course of COVID-19. Coupled with the toxic effect of the SARS-CoV-2 virus on the myocardium, the disease may lead to progressive heart failure and poor outcomes.

## 1. Introduction

In recent years, cardiac amyloidosis (CA) has become increasingly recognized as a significant cause of heart failure (HF) among elderly patients [1,2,3]. Postmortem studies conducted over the past several decades indicate that the prevalence of concealed CA reaches 25% in adults >80 years old and 32% of patients >75 years old who have HF with preserved ejection fraction (HFpEF) [4].

Transthyretin amyloidosis (ATTR) is one of the forms of CA. In transthyretin amyloid cardiomyopathy (ATTR-CM), misfolded transthyretin (TTR) proteins deposit in the heart muscle. TTR is a 55 kDa tetrameric protein transport protein, primarily synthesized by the liver that functions to carry thyroxine molecules and binds to retinol-binding protein [5,6,7].

ATTR pathogenesis is linked to the destabilization of normal TTR tetramer structure. As a result, the tetramers dissociate into monomers which then misfold and aggregate into insoluble amyloid fibrils [8,9,10].

ATTR is categorized into two main types: an acquired condition associated with the abnormal buildup of wild-type transthyretin (ATTRwt) and variant (ATTRv), a hereditary form caused by mutations in the TTR gene [11,12].

So far, around 150 mutations in the transthyretin (TTR) gene have been identified, leading to a variety of clinical phenotypes. These include primary polyneuropathy (V30M mutation); cardiomyopathy (linked to mutations like V20I, V122I, L111M, and I68L); and mixed phenotypes (E89Q and T60A mutations) [13,14].

The most common amyloidogenic mutation worldwide is V122I, and it is primarily associated with familial amyloid cardiomyopathy [15,16].

Both variants of transthyretin cardiomyopathy are characterized by a rapid and progressive decline in heart function, and standard heart failure medications may not provide complete or partial response in such patients [17].

In elderly patients, it can be challenging to diagnose amyloidosis due to the absence of specific symptoms and its ability to mimic other existing chronic conditions, especially in the early stages. This can significantly reduce the opportunity for timely diagnosis and early intervention with disease-modifying therapies [18,19,20].

Patients with ATTRwt amyloidosis represent a population particularly vulnerable to COVID-19 morbidity. They have a higher risk of critical and severe COVID-19 outcomes due to age and underlying heart failure [21]. This retrospective study is focused on histopathological analysis of autopsy specimens taken from patients with clinically underdiagnosed ATTR amyloidosis, mostly affecting the heart, who died from severe course of COVID-19.

## 2. Results

We evaluated the results of gross and microscopic findings in the myocardium in 19 patients with pre-existing transthyretin amyloidosis who died from severe COVID-19. The study included 10 males and 9 females aged 80 to 97 years (mean age of all patients—90; men—90.3, women—89.3 years). The duration of hospitalization (from admission to death) ranged from 1 to 19 days. Data on the primary diagnosis, causes of deaths, and comorbidities are given in Table 1.

Pre-mortem PCR tests for detecting SARS-CoV-2 RNA were performed in all cases, and the results showed that all 19 tested patients had COVID-19 infections. Tests performed on autopsy specimens, specifically from the myocardium and lungs, were positive for SARS-CoV-2 RNA in 10 (53%) of 19 cases.

### 2.1. Gross and Microscopic Findings in the Myocardium

On gross examination, a heart weight ranged from 310 to 640 g. The dilation of cardiac cavities was observed in 16 (84%) of 19 deceased patients. The thickness of the left ventricular wall varied from 1.2 to 2.5 cm and that of the right ventricular wall from 0.2 to 1.0 cm. Hypertrophy of the left and right ventricle was revealed in 17 (89%) and 12 (63%) patients, respectively. On gross section, in 8 (42%) cases, the heart muscle appeared stiff and brown with fibrous interlayers. In 11 (58%) deceased individuals, the heart muscle appeared flaccid, and in some cases, the myocardium contained pronounced fibrosis and small scars. Metabolic myocardial necroses were determined in 4 (21%) patients, cor pulmonale in 5 (26%), and hydropericardium in 2 (11%) cases.

**Figure 1:**
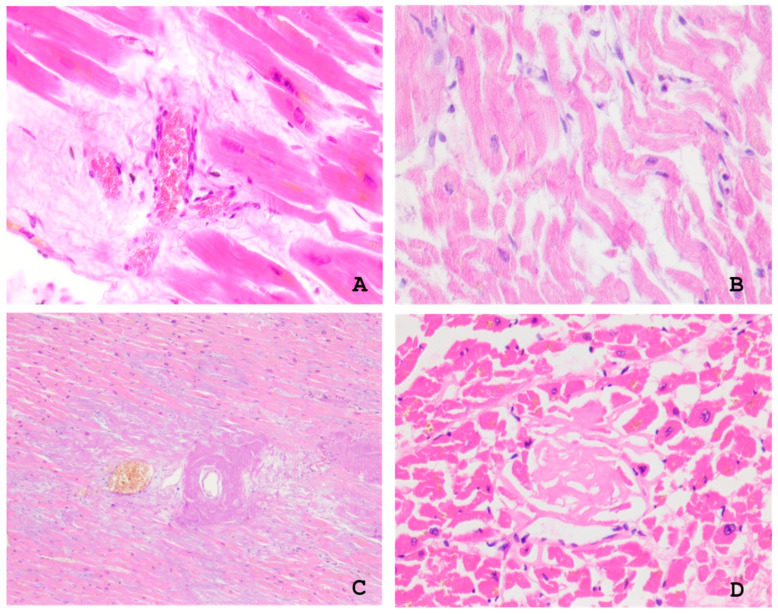
(**A**)—proliferation of vascular endothelial cells amid venous plethora with pronounced perivascular edema and sclerosis, ×200; (**B**)—wave-like deformation of myofibrils in cardiac muscle cells, ×100; (**C**)—massive perivascular amyloid deposits associated with venous plethora, ×40; (**D**)—homogenous eosinophilic amyloid deposits arranged in a reticular pattern with fragmented cardiac muscle cells, ×100.

Microscopic examination of the myocardium revealed focal perivascular and interstitial sclerosis, fragmentation and focal necrosis of cardiomyocytes, sclerosis, and hypertrophy of the blood vessel walls. Microvascular dysfunction was observed in 12 (63%) autopsy cases and plethora with proliferation of vascular endothelial cells (endothelial swelling and proliferation) in 7 (37%) (Figure 1). Other autopsy findings included focal cardiosclerosis and cardiomyocyte dissociation in 15 (68%) cases and hypertrophy and atrophy of cardiomyocytes in 17 (77%) and 7 (32%) cases, respectively. The autopsy proved the presence of myocarditis manifestations in 4 (18%) cases. Other findings included wave-like deformations of the muscle fibres, which can be considered an indicator of decompensated heart failure (Figure 1).

This section may be divided by subheadings. It should provide a concise and precise description of the experimental results, their interpretation, as well as the experimental conclusions that can be drawn.

**Figure 2:**
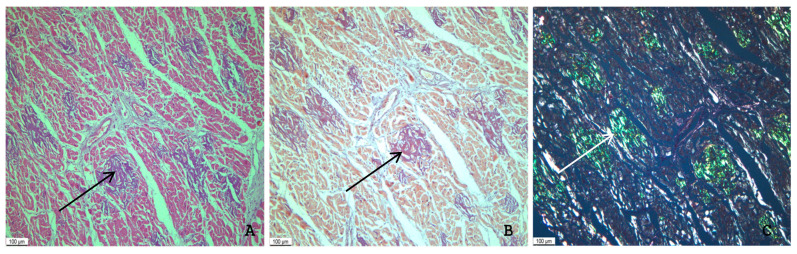
An example of a microscopic image of cardiac tissue of the deceased male patient with ATTR amyloidosis and COVID-19. Homogenous eosinophilic amyloid deposits (black arrow), H&E staining (**A**). Brick-red coloration of amyloid deposits (black arrow) after Congo red staining (**B**). Birefringence of amyloid structures (white arrow) under polarized light microscopy (**C**), ×40.

Amyloid deposits appeared as a homogenous, acellular, eosinophilic material which showed positivity with Congo red stain and exhibited characteristic birefringence under polarized light microscopy (Figure 2). A solely interstitial amyloid deposition was observed in 13 (68%) autopsies, while both interstitial and intravascular amyloid deposits were detected in 6 (32%) cases. A reticular pattern of interstitial amyloid deposits was determined in 5 (26%) cases and a macro- and micronodular pattern in 6 (32%), and in 8 (42%) cases the amyloid deposits were arranged in a combined (reticular and nodular) pattern.

**Figure 3:**
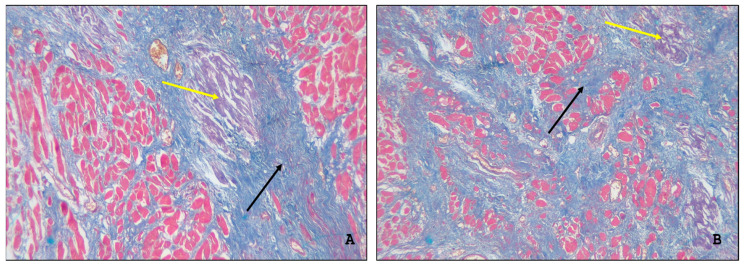
The postmortem microscopic examination of the myocardial specimens. (**A**,**B**) Interstitial pattern of amyloid deposition (yellow arrow) associated with large cardiosclerosis foci (black arrow). Mallory staining, ×100.

According to clinical records, seven patients had rapidly progressive heart failure resistant to drug therapy. The postmortem microscopic examination of the myocardial specimens taken from these patients revealed multiple sites of interstitial amyloid deposits associated with focal cardiosclerosis (Figure 3A,B).

To assess the contribution of transthyretin amyloidosis to the severity of clinical course of COVID-19, we performed postmortem examination of myocardial samples of 10 patients (a control group) who died from the progressive complications of SARS-CoV-2 infection. The control group consisted of six female and four male patients aged 58–92 years (mean 76.8 years). The duration of their hospitalization from the date of admission to the date of death ranged from 3 to 11 days (mean 5.2 days). In nine patients, COVID-19 was the primary diagnosis, and the immediate cause of death was the progressive respiratory failure related to the acute respiratory distress syndrome of adults. In one case, the primary diagnosis was chronic cerebral ischemia with later-onset cerebral edema and bilateral hydrothorax.

**Figure 4:**
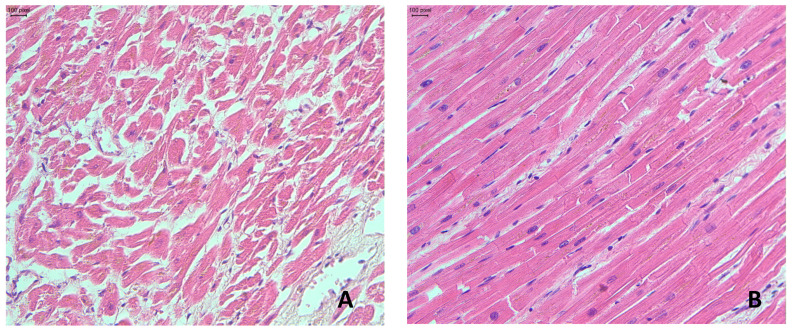
Histopathological changes in the myocardium: (**A**,**B**)—hypertrophy of cardiomyocytes with a moderate edema in *intracellular* compartments and pericellular fibrosis (H&E staining), ×100.

Histopathological findings in the myocardium in all cases included dissociation and hypertrophy of cardiomyocytes with perinuclear lipofuscin granules, interstitial and perivascular edema, and diffuse myocardial fibrosis (Figure 4).

Histopathological analysis has demonstrated that morphological changes in the myocardium were more intense and widespread in patients with co-existing transthyretin amyloidosis and COVID-19 than those in patients without amyloid deposits. In patients with co-existing ATTR and COVID-19, autopsy findings included diffuse interstitial amyloid deposits, significant hypertrophy of cardiomyocytes, interstitial and focal cardiosclerosis, and microcirculatory disturbances. In the control group of patients, myocardial lesions were characterized by moderate cardiomyocyte hypertrophy, intercellular edema, focal dystrophy, and small foci of fibrosis.

### 2.2. Immunohistochemical Typing of Amyloid

**Figure 5:**
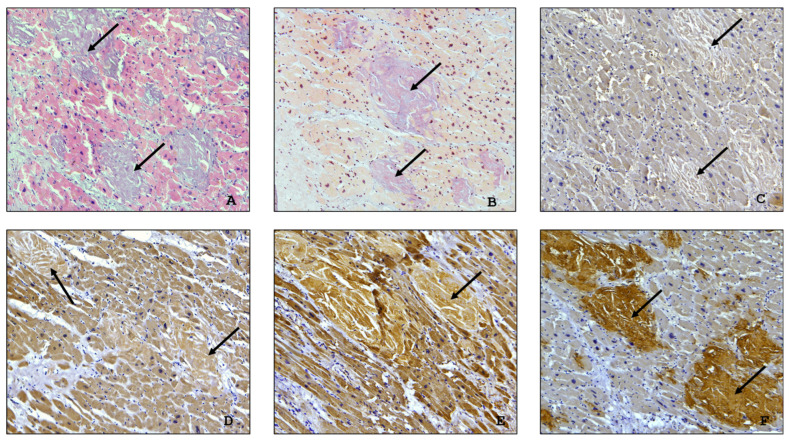
The myocardium of patient C., 86 years old, with ATTR amyloidosis (an autopsy specimen). Interstitial pattern of homogenous acellular eosinophilic amyloid deposits (black arrow) after H&E staining (**A**). Brick-red coloration of amyloid deposits after Congo red staining (**B**). A negative IHC reaction with an antibody against AA amyloid (**C**) and AL lambda amyloid (**D**). A weak background IHC reaction with an anti-AL kappa amyloid antibody (**E**). A positive IHC reaction with an antibody against ATTR (**F**). (**A**–**F**) ×100.

The diagnosis of ATTR amyloidosis was confirmed in all 19 cases through immunohistochemical (IHC) typing of amyloid. A weak positive immunostaining with anti-κ-light chain antibody was observed in two cases. However, it was interpreted as a false positive reaction, taking into consideration a strong anti-transthyretin antibody immunostaining of the deposited amyloid (Figure 5).

### 2.3. Assessment of the Intensity of Myocardial Damage by Amyloid

**Figure 6:**
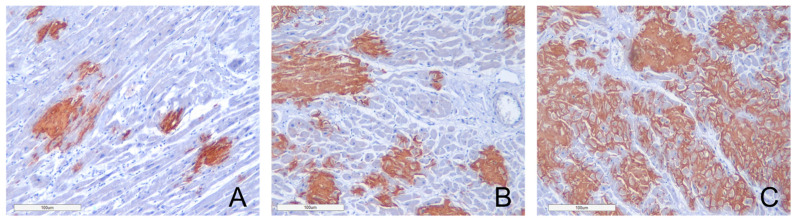
The degrees of amyloid deposition in the myocardium. (**A**)—Grade 1 (0–20% 20% of amyloid deposits); (**B**)—Grade 2 (20–40% of amyloid deposits); (**C**)—Grade 3 Grade 3 (40% and over). Amyloid is stained with polyclonal antibody to Transthyretin, ×100.

To assess the intensity of amyloid deposits in myocardium, a grading system was used with three grades of amyloid depositions (Grade 1—0–20% in the field of vision; Grade 2—20–40%; Grade 3—40% and over). Our findings demonstrate that Grade 1 amyloid depositions were identified in 6 (31.6%) cases, Grade 2 depositions were recorded also in 6 (31.6%) cases, and Grade 3 depositions were detected in 7 (36.8%) deceased patients’ findings (Figure 6). We found out that the mean duration of hospitalization (from admission to death) in patients with Grade 1 (less than 20% of amyloid deposition in the field of vision) was 10 days, in patients with Grade 2 (20–40%) 7.2 days, and in patients with Grade 3 (40% and over) 5 days.

Thus, we can conclude that in the deceased patients with intense amyloid deposits (Grade 3), the duration of hospitalization (from admission to death) was two-fold shorter than in patients with Grade 1 of amyloid deposition. These findings illustrate a more severe clinical course of COVID-19 in patients with massive amyloid deposition.

The available histopathological findings and clinical characteristics (the age range of 80 to 97 years, an equal number of male and female patients, no known family history of amyloidosis, and the focal interstitial pattern of amyloid deposition, primarily in the myocardium and blood vessel walls) are highly consistent with the classical diagnostic criteria of sporadic ATTRwt amyloidosis.

Our histopathological findings suggest that patients with pre-existing sporadic ATTRwt and contracted COVID-19 have significant structural changes in the heart. In addition to common manifestations of cardiac amyloidosis, such as cardiomegaly, hypertrophy and diffuse amyloid deposits, these patients had signs of acute myocardial injury.

The occurrence of COVID-19 in persons with underlying cardiac amyloidosis may lead to rapidly progressive heart failure. Our data emphasize that a broader screening for amyloidosis in cardiology practice is crucial, especially in elderly patients who experience symptoms of hypertrophic restrictive cardiomyopathy.

## 3. Discussion

Pre-existing cardiovascular disease significantly increases the morbidity of COVID-19 and is strongly associated with poor outcomes. Higher mortality rates are reported in patients with chronic heart failure, ischemic heart disease, postinfarction cardiosclerosis, and arterial hypertension [22,23,24,25]. While ATTR-CM is an uncommon and often underdiagnosed condition, it can significantly impact the clinical course of COVID-19 [16].

Currently, there is a lack of published scientific data specifically describing histopathological myocardial lesions in patients with co-existing ATTRwt and SARS-CoV-2 infection. Studies examining heart muscle damage in patients with such comorbidities are indeed scarce, and, as a rule, they represent incidental autopsy findings or case studies. The research was not focused on detailed histopathological analysis of cardiac injury in this specific combined context. For instance, in the study of Haslbauer et al., a retrospective cardiopathological analysis of the heart from 23 autopsies of COVID-19 patients was carried out, and in six cases (26%), ATTRwt was found as a comorbidity. However, this comorbidity was not explored in detail within the published paper [26].

The study of Ferrer-Gómez A. et al. presents autopsy findings in cardiac samples of 30 patients who died due to severe COVID-19. In this group of patients, only one case of ATTRwt was detected [27]. Mikhaleva L. et al. performed postmortem examination in 22 patients with different types of systemic amyloidosis and severe clinical course of COVID-19. ATTRwt was identified in eight cases (36%), and in all these cases, it was diagnosed only at the autopsy [28].

Menter et al. in 2020 reported the autopsy findings of 21 patients who died from severe COVID-19. Six of these patients (28.5%) were diagnosed with cardiac amyloidosis of ATTRwt type at postmortem examination. The researchers noted that in this patient cohort, the prevalence of ATTRwt has reached statistical significance. This finding differed from the ATTRwt prevalence revealed in the autopsies performed in the same hospital settings in 2018 and 2019—22 cases (6.4%) of 345 autopsies [29]. The presented data can be considered a poor prognostic indicator for patients with these comorbidities.

Our study has compiled more data than any previous study in the field, as it provides the most comprehensive description of autopsy findings in the myocardium of 19 patients with co-existing COVID-19 and ATTRwt. The study highlights histopathological characteristics of heart damage resulting from the combined effects of these two severe conditions.

Various mechanisms have been suggested to explain the increased vulnerability of patients with underlying ATTR-CM for COVID-19 disease, including specific morphological and functional changes in the myocardium. Amyloid deposits in the myocardium cause thickening and stiffening of the ventricular walls, reduced systolic output, and a chronic increase in diastolic pressure. In such patients, even moderate physical stresses like tachycardia, hyperthermia, or metabolic stress from an infection can cause a rapid worsening of hemodynamics and the development of acute heart failure [30].

Patients with underdiagnosed amyloid cardiomyopathy and COVID-19 represent a population particularly vulnerable to fatal heart failure. They might require specialized therapeutic management for COVID-19, taking into consideration the underlying disease.

The altered activity of immune system in patients with amyloidosis contributes to the severe clinical course of COVID-19. According to scientific studies, some patients with ATTR-CM may exhibit persistently elevated levels of pro-inflammatory cytokines like interleukin-6 (IL-6) and tumour necrosis factor-alpha (TNF-α), even in the absence of a disease relapse. In this context, Hein SJ et al. assessed the prognostic value of IL-6 in cardiac ATTR amyloidosis. They found that IL-6 levels from wild-type ATTR patients were significantly elevated compared to healthy controls and correlated with clinical presentation of ATTR-CM [31].

Myocardial damage in COVID-19 is linked to multiple factors, including cytopathic effect of the virus, altered endothelial function, and an overactive inflammatory response. SARS-CoV-2 infection can significantly impact the hearts of individuals with pre-existing amyloidosis, particularly those with structural cardiac changes and heightened functional vulnerability.

Our histopathological findings confirm that patients with pre-existing ATTR and contracted COVID-19 had severe cardiovascular sequelae. The revealed massive interstitial amyloid deposits in the myocardium coupled with focal cardiosclerosis, underlying the virus-induced cardiomyocyte damage, may contribute to the development of cardiac complications and acute heart failure. Along with hypoxemia and hypercoagulation, these complications could ultimately result in multiorgan failure.

In our study, ATTR amyloidosis was found with equal frequency in male and female patients, and in all cases the diagnosis was established only at autopsy. The study of Muchtar et al. indicates that most of patients with clinically diagnosed ATTR are men, although the prevalence of females is higher in autopsy series [32].

Though research data involving patients with rare health conditions remain scarce, the findings suggest that amyloidosis patients are likely to be at a higher risk for COVID-19 mortality compared with the general population. Lewis E et al. reported an excess of deaths of 128% among the ATTR cohort when comparing pre-pandemic years 2018 and 2019 with the pandemic years of 2020 and 2021 [33].

In general, patients with pre-existing ATTR appear to be at higher risk for morbidity and mortality from COVID-19. Although it is predominantly a respiratory illness, COVID-19 contributes to cardiovascular and systemic complications in this patient cohort. Authors should discuss the results and how they can be interpreted from the perspective of previous studies and of the working hypotheses. The findings and their implications should be discussed in the broadest context possible. Future research directions may also be highlighted.

## 4. Materials and Methods

The study describes autopsy findings in 19 patients with severe clinical course of novel coronavirus disease and pre-existing transthyretin cardiac amyloidosis (10 men and 9 women aged 80 to 97 years). These cases were retrospectively selected from the Amyloid Registry of Avtsyn Research Institute of Human Morphology of Federal State Budgetary Scientific Institution “Petrovsky National Research Centre of Surgery.”

Pre- and post-mortem examinations of the collected samples for SARS-CoV-2 RNA using real-time PCR were performed in all cases. For microscopy, the samples were fixed with 10% neutral buffered formalin solution and paraffin embedded. All histological sections were stained with H&E and Congo red and then examined under polarized light. Immunohistochemical amyloid typing was performed with a broad panel of antibodies: polyclonal antibody against amyloid P-component (Anti-Serum Amyloid P/SAP antibody, Abcam, Cambridge, UK); polyclonal antibody against transthyretin (Prealbumin, Cloud-Clone Corp., Katy, TX, USA); monoclonal antibody against AL kappa (Anti-Kappa light chain antibody, clone CH15, Leica Biosystems, Novocastra Newcastle Upon Tyne, UK™) and AL lambda (Anti-Lambda light chain antibody, clone SHL53, Leica Biosystems, Newcastle Upon Tyne, UK™) light chains; and against AA amyloid (clone C3, Cloud-Clone Corp.) using the Leica BOND-MAX stainer (Germany).

To evaluate the intensity of amyloid deposits, a semi-quantitative grading system was used with the following three grades of amyloid deposition: Grade 1—amyloid deposition made up less than 20% in the field of vision; Grade 2—20–40%; Grade 3—40% and over. We used these grading criteria to assess the extent of cardiac involvement. The determined grade could play an important role in clinical settings, as the administered therapy may become less effective, if amyloid deposition comprises more than 20% in the field of vision [34].

## 5. Conclusions

Thus, patients with ATTR amyloidosis represent a population with increased vulnerability to COVID-19 morbidity. At the same time, poor outcomes of COVID-19 are more likely in patients with underlying sporadic ATTRwt amyloidosis due to the development of heart failure and old age.

Underdiagnosed amyloidosis in patients admitted to ICU can lead to inappropriate treatment decisions, and significantly increases the risk of severe complications, potentially causing fatal outcomes.

When elderly patients present with signs of cardiosclerosis, physicians should consider and rule out sporadic ATTRwt, as these comorbidities can induce a progressive heart failure.

## Figures and Tables

**Table 1:** Clinical and morphological characteristics of patients.

Patient No.	Gender	Age (Years)	Primary Diagnosis	Immediate Cause of Death	Comorbidities	Length of Hospital Stay (Days)	Intensity of Amyloid Deposits in the Heart *
1	M	83	SARS-CoV-2 infection (COVID-19)	Acute respiratory distress syndrome	Primary hypertension involving the kidneys. Cardiac amyloidosis	11	Grade 2
2	M	88	SARS-CoV-2 infection (COVID-19)	Acute respiratory distress syndrome	Diffuse small-focal cardiosclerosis Cardiac amyloidosis	3	Grade 3
3	F	80	SARS-CoV-2 infection (COVID-19)	Acute respiratory distress syndrome	Primary hypertension Amyloidosis involving the heart and lungs	19	Grade 1
4	M	85	SARS-CoV-2 infection (COVID-19)	Acute respiratory distress syndrome	Amyloidosis involving the heart, hepatic and pulmonary vessels	8	Grade 3
5	M	87	SARS-CoV-2 infection (COVID-19)	Acute respiratory distress syndrome	Diffuse small-focal cardiosclerosis. Cardiac amyloidosis	7	Grade 1
6	M	95	Postinfarction cardiosclerosis	Myocardial infarction	SARS-CoV-2 infection (COVID-19) Cardiac amyloidosis	1	Grade 2
7	M	95	SARS-CoV-2 infection (COVID-19)	Acute respiratory distress syndrome	Aortic atherosclerosis (Grade 3, Stage IV). Cardiac amyloidosis. Gallstone disease: chronic cholecystitis. Kidney cysts. Chronic inactive tubulointerstitial nephritis	11	Grade 3
8	F	97	Postinfarction cardiosclerosis.	Myocardial infarction	SARS-CoV-2 infection (COVID-19) Cardiac amyloidosis	6	Grade 2
9	F	92	SARS-CoV-2 infection (COVID-19)	Cardiopulmonary decompensation	Cerebral atherosclerosis, encephalopathy. Cardiac amyloidosis	1	Grade 3
10	F	90	Postinfarction cardiosclerosis	Myocardial infarction	SARS-CoV-2 infection (COVID-19) Cardiac amyloidosis	4	Grade 1
11	M	94	SARS-CoV-2 infection (COVID-19)	Acute respiratory distress syndrome	Diffuse small-focal cardiosclerosis. Left atrial appendage thrombosis. Cardiac amyloidosis	8	Grade 2
12	M	88	SARS-CoV-2 infection (COVID-19)	Acute respiratory distress syndrome	Diffuse small-focal cardiosclerosis. Cardiac amyloidosis	2	Grade 3
13	F	85	Infective endocarditis of the mitral valve. Atherosclerotic foot gangrene	Sepsis. Metabolic myocardial necrosis	SARS-CoV-2 infection (COVID-19). Cardiac amyloidosis. Gallbladder cancer with metastases in lymph nodes of the hepatic hilum and lesser gastric curvature	2	Grade 1
14	F	89	Postinfarction cardiosclerosis	Myocardial infarction	SARS-CoV-2 infection (COVID-19) Cardiac amyloidosis	5	Grade 2
15	F	93	SARS-CoV-2 infection (COVID-19)	Acute respiratory distress syndrome	Cerebral atherosclerosis, encephalopathy. Amyloidosis affecting the heart, kidneys, pancreas, and lungs	11	Grade 1
16	M	94	SARS-CoV-2 infection (COVID-19)	Acute respiratory distress syndrome	Chronic cerebral ischemia. Cardiac amyloidosis. Arteriolar nephrosclerosis	17	Grade 1
17	F	89	Ischemic infarction in the left parietotemporal area	Cerebral edema	SARS-CoV-2 infection (COVID-19) Cardiac amyloidosis	2	Grade 3
18	M	94	SARS-CoV-2 infection (COVID-19)	Acute respiratory distress syndrome	Primary hypertension. Cardiac amyloidosis	12	Grade 2
19	F	89	Postinfarction cardiosclerosis	Myocardial infarction	SARS-CoV-2 infection (COVID-19) Cardiac amyloidosis	8	Grade 3

* Grade 1: amyloid deposition made up less than 20% in the field of vision; Grade 2: from 20 to 40%; and Grade 3: 40% and over.

## Data Availability

All data and materials are available upon reasonable request. Address to Z.G. (email: gioeva_z@mail.ru) or L.M. (email: mikhalevalm@yandex.ru), Avtsyn Research Institute of Human Morphology of Federal State Budgetary Scientific Institution “Petrovsky National Research Centre of Surgery,” Moscow, Russian Federation.

## Abbreviations

The following abbreviations are used in this manuscript:

ATTR	Transthyretin amyloidosis
ATTR-CM	Transthyretin amyloid cardiomyopathy
ATTRwt	Wild-type transthyretin amyloidosis
ATTRv	Variant transthyretin amyloidosis
HF	Heart failure
COVID-19	Coronavirus-19 disease
H&E	Hematoxylin and eosin
IHC	Immunohistochemistry
